# Reproductive and Developmental Nanotoxicity of Carbon Nanoparticles

**DOI:** 10.3390/nano12101716

**Published:** 2022-05-17

**Authors:** Drahomira Holmannova, Pavel Borsky, Tereza Svadlakova, Lenka Borska, Zdenek Fiala

**Affiliations:** 1 Institute of Preventive Medicine, Faculty of Medicine in Hradec Kralove, Charles University, 50003 Hradec Kralove, Czech Republic; holmd9ar@lfhk.cuni.cz (D.H.); svadlakovat@lfhk.cuni.cz (T.S.); borka@lfhk.cuni.cz (L.B.); fiala@lfhk.cuni.cz (Z.F.); 2 Institute of Clinical Immunology and Allergology, University Hospital and Faculty of Medicine in Hradec Kralove, Charles University, 50003 Hradec Kralove, Czech Republic

**Keywords:** carbon nanoparticles, graphene, MWCNT, nanotoxicity, reproduction

## Abstract

The presented review aims to summarize the knowledge regarding the reproductive and developmental toxicity of different types of carbon nanoparticles, such as graphene, graphene oxide, multi- and single-walled nanotubes, fullerenes, and nanodiamonds. Carbon nanoparticles have unique chemical and physical properties that make them an excellent material that can be applied in many fields of human activity, including industry, food processing, the pharmaceutical industry, or medicine. Although it has a high degree of biocompatibility, possible toxic effects on different tissue types must also be taken into account. Carbon nanoparticles are known to be toxic to the respiratory, cardiovascular, nervous, digestive system, etc., and, according to current studies, they also have a negative effect on reproduction and offspring development.

## 1. Introduction

Carbon is one of the most common and most important elements in our universe. It is an essential component of macromolecules indispensable for life, such as proteins, lipids, nucleic acids, and carbohydrates with unique chemical and physical properties. These extraordinary chemical-physical properties are further enhanced in carbon-based nanomaterials. Carbon-based nanomaterials are characterized by excellent electrical and heat conductivity, extreme stiffness, strength, toughness, and high biocompatibility and low toxicity [1].

Carbon nanomaterials have found applications in various industries, including electronics, agriculture, food, pharmaceuticals, medicine, and cosmetics [2,3,4,5]. Among the most widely used and studied carbon nanoparticles are graphene, graphene oxide, carbon nanotubes, fullerenes, and nanodiamonds. Graphene is a two-dimensional (2D) material, a monolayer where carbon atoms are arranged in a honeycomb lattice structure. Graphene has unique properties, including high thermal and electrical conductivity and stability, high flexibility and elasticity, hardness and resistance, and large surface area [6].

Graphene oxide (GO) and reduced graphene oxide (rGO) are graphene derivates with different structural and chemical properties. GO is usually synthesized by chemical oxidation and exfoliation of graphite. It possesses various oxygen groups (hydroxyl, carboxyl, epoxy groups) that functionalize the surface and modify the properties of CNPs. To reduce GO, chemical, thermal, or photo-thermal reduction can be used; however, rGO did not reach the original structure of pristine graphene and still contains residual oxygen, even after strong reduction. The main functional differences between GO and rGO are in electrical conductivity. GO shows low electrical conductivity and lower mechanical strength compared to rGO and pristine graphene [7,8]. Carbon nanotubes are CNPs with tubular structure; graphene sheets rolled into a cylindrical shape. They are classified on the basis of the number of walls, such as single-walled, double-walled, or multi-walled. Like graphene, nanotubes have exceptional properties, especially high electrical and thermal conductivity, strength and elasticity, and large surface that can be easily functionalized [9,10].

Fullerenes (buckyballs) are a class of carbon allotropes with a spherical or ellipsoidal shape. Their size is dependent on the number of carbons—C_60_, C_70_, C_80_, etc. The most common fullerene structure C_60_ is a molecule that consists of 60 carbon atoms arranged as 20 regular hexagons and 12 regular pentagons. Fullerenes are soluble in organic solvents, especially C_60_, C_70_, and easily functionalized [11]. Interestingly, fullerenes are great antioxidants. The antioxidant capacity can be enhanced by functionalization with hydroxyl molecules [12,13,14].

Very popular CNPs are nanodiamonds that consist of a diamond core (sp^3^ bonded carbon atoms) and amorphous carbon layers (sp^2^ layers covering the core). Nanodiamonds are chemically inert, have high hardness, optical transparency, and thermal conductivity. Importantly, the large surface of nanodiamonds is functionalized with various functional groups (hydroxyl, carboxyl, ether, ketone) [15]. The oxygen functional groups exhibit significant affinity for water and polar organic solvents. Conversely, they do not have affinity for oils or non-polar solvents, therefore nanodiamonds form agglomerates in these solutions [16].

Graphene quantum dots (GQDs) are quasi-spherical nanoparticles that are produced by destroying larger carbon materials, such as carbon nanotubes, graphene or GO sheets or carbon fibers by strong acid oxidation, hydrothermal or solvothermal treatment, etc. GQDs have remarkable photochemical and photoluminescent properties. GQDs can be dissolved in most polar solvents without additional chemical treatments and have excellent stability compared to other fluorescent dyes, making GQDs suitable for use in bioimaging [17,18].

Although the positive properties of carbon nanoparticles are well known and have potential to be used in biomedicine, their potential negative effects on health are not yet well understood, and there are growing concerns about the safety of carbon nanomaterials [19,20].

Very critical is toxicity, which affects the reproductive system and can harm future generations.

The reproductive system of males and females is composed of many organs that are differently sensitive to potentially damaging factors and substances. Reproductive/developmental toxicity due to the OSHA Hazard Communication Standard (HCS 2012) is defined as a substance-induced adverse effect (a) on sexual function and fertility and (b) on the development of offspring during intrauterine development, immediately after birth, or postnatally (disorders of further development and growth) [21,22].

Impairment of fertility in both sexes includes changes in sexual behavior, structural and functional changes in the male and female reproductive systems. Changes can disrupt one or more stages of fertilization up to the point of embryo implantation in the uterus. Specifically, it is endocrine dysfunction and changes in the processes of gametogenesis, libido, mating behavior, fertilization, transport of fertilized eggs through a fallopian tube to the uterus, and implantation into the endometrium [23].

It is generally accepted that the embryo and fetus are more sensitive to xenobiotics than the fully developed organism and therefore determining the developmental toxicity of carbon nanoparticles (CNP) is essential, especially when there is the possibility that these particles can cross the placenta and exhibit cytotoxicity toward different cells and tissue types [24,25].

## 2. Reproductive Toxicity In Vitro Studies

Several in vitro toxicity studies evaluated the deleterious effect of CNP on cells of the reproductive system, especially sperm and oocytes.

First, we mention the negative effect of multi-walled carbon nanotubes on steroidogenesis and the production of sex hormones, which are essential for reproduction (gametogenesis, ovulation, and sexual behavior). Qu et al. showed that multi-walled carbon nanotubes (MWCNTs) inhibited progesterone production in preovulatory rat granulosa cells. Production decreased significantly at a concentration of 10 and 50 μg/mL/48 h. MWCNTs altered the expression of steroidogenic proteins StAR that transfer cholesterol from the outer to the inner mitochondrial membrane, where cholesterol is metabolized by P450scc to pregnenolone. The inhibitory effect is reversible. Removal of MWCNTs restored progesterone production. Furthermore, MWCNTs induced ROS production and slightly altered mitochondrial membrane potential. These results suggest that steroidogenesis is compromitted by StAR inhibition and oxidative stress induced by MWCNTs, which act as an endocrine disruptor [26].

Male germ cells are susceptible to xenobiotics, including CNPs. Gurunathan et al. determined the toxic effect of graphene oxide (GOs) on Leydig and Sertoli cells (TM3 and TM4). They exposed cells to two types of GOs with different lengths (20 and 100 nm) and different zeta potential (electrokinetic potential in colloidal systems). Both GOs inhibited cell viability and proliferation in a dose-dependent manner, induced the release of lactate dehydrogenase (a marker of cellular damage or death), and altered mitochondrial membrane potential which was associated with elevated reactive oxygen species (ROS). Furthermore, GOs were responsible for DNA damage depending on nucleoside oxidation, and 8-oxo-dG formation, and suppression of pro-apoptotic gene expression (Bax, Bak, p53, p21, caspase-3), while the expression of genes coding anti-apoptotic proteins (Bcl-2) increased. Cell survival was also altered by suppression of EGFR (epidermal growth factor receptor) and AKT kinase phosphorylation. Interestingly, shorter GOs caused significantly greater damage in some measured parameters, and TM3 appeared to be more susceptible to the GOs exposure [27].

Ji et al. exposed TM4 and GC-2 spd (mouse testicular germ cell lines) to GO quantum dots (QD). Although graphene oxide quantum dots (GOQDs) did not affect cell viability, they induced apoptosis in both cell lines. Transmission electron microscopy images showed that GOQD treatment increased the number of autophagosomes, and thus autophagy. The degradation of sequestered material in autophagosomes depends on the fusion between autophagosomes and lysosomes. The authors found that this process was not inhibited, but that undegraded cargo occurred. This phenomenon was due to a reduction in lysosomal activity and the ability to degrade the material. The accumulation of undegraded cargo is a hallmark of aging and senescent cells [28].

GC-2 spd cells are sensitive not only to GOQD but also to MWCNTs. The study by Xu et al. showed that while the dose of 0.5 μg/mL MWCNTs was not lethal to cells, the accumulation of MWCNTs in mitochondria was detected. The presence of MWCNTs in mitochondria was associated with a decrease in the expression of mitochondrial-related genes, the rate of oxygen consumption, and especially with decreased ATP production that is necessary to maintain cell function and survival [29].

The toxic effect of CNPs was evaluated not only on mouse germinal cells but also on the spermatozoa of buffalos or boars. The toxicity of MWCNTs was tested by Sanand et al. in buffalo spermatozoa and the half-maximum inhibitory concentration (IC_50_) was determined. Buffalo sperm were exposed to different doses of MWCNT for 30, 60, and 120 min. MWCNTs time- and dose-dependently decreased cell viability, severely depressed the membrane integrity, increased malondialdehyde levels (a marker of oxidative stress), and decreased the activity of antioxidant enzymes (glutathione peroxidase /GTP/, superoxide dismutase /SOD/) [30].

The results confirming the toxicity of CNPs were obtained in a study by Bernabò et al. who exposed boar spermatozoa to GO at different concentrations. Although doses of 5, 10, and 50 μg/mL were toxic to cells (reduced viability, fertilization capacity, and impaired acrosome integrity and adhesion capacity), lower doses of 0.5 and 1 μg/mL promoted sperm fertilization capacity. Importantly, the authors demonstrated that GO interacted with sperm membranes and altered membrane fluidity by extracting cholesterol from the membrane [31].

Li et al. observed quite different effects of CNPs when exposed boar spermatozoa to carboxylated fullerene (C_60_–COOH). Sperm incubation with C_60_–COOH at a dose of 2 μg/mL for 10 days increased sperm motility compared to the control group, improved acrosome integrity and mitochondrial activity, and reduced oxidative stress. C60 has been shown to have antioxidant effects [32].

The conclusions of studies on human sperm are still not concise. Asghar et al. described that carboxylated single-walled carbon nanotubes (SWCNT–COOH) induced ROS production of ROS in human spermatozoa at a concentration of 25 μg/mL, while the presence of reduced GOs did not increase oxidative stress. Importantly, neither GOs nor SWCNTs at a dose of 1–25 μg/mL affected sperm viability [33]. Human sperm, in the study by Aminzadeh et al., were exposed to SWCNT–COOH or MWCNT–COOH (0.1–100 μg/mL/5 h). Both CNPs did not attenuate sperm viability. However, sperm motility decreased in a dose-dependent manner, and even the lowest concentration of nanoparticles increased ROS production, which may be associated with mitochondrial and DNA damage [34].

As follows, exposure to female germ cells induces several serious changes. The results showed that the oocytes are sensitive to CNP treatment. Lin et al. documented that graphene QDs altered the maturation of mouse oocytes. Oocytes incubated with graphene QD doses failed to extrude polar bodies. This effect was dose-dependent and was accompanied by accumulation of intracellular ROS and DNA damage. Furthermore, graphene QDs were detected in oocytes, located primarily in the nucleus and near the mitochondria, whose morphology and functions were severely altered [35].

The resumption of rat oocyte meiosis was described by Lei et al. using an in vitro maturation culture model and exposing oocyte granulosa cells (OGCs) to fullerenols. Fullerenols reduced transzonal protrusions (TZPs), accelerating the retraction of TZPs from oocytes. Transzonal projection is the connection between granulosa cells (the cell layer surrounding the oocyte) and oocytes and forms a functional complex that is essential for the maintenance and development of oocytes. All doses reduced TZPs and only a few thin filaments of granulosa cells connected to the oocyte (in the control culture, the filaments were intact and abundant). Furthermore, fullerenols reduced the expression of connexin 43 in granulosa cells, which is a part of gap junctions. The two-hour treatment decreased expression by 56%. Retraction of TZPs and lower expression of connexin 43 altered gap junction channels and reduced mass transport, leading to a decrease in cyclic adenosine monophosphate in oocytes and an accelerated resumption of meiosis, which can lead to reduced oocyte quality [36].

While there is a possibility that fullerenols have cytotoxic potential, in the field of genotoxicity, they have a rather protective effect. Mrdanovic et al. found that fullerenols, even at high concentrations, reduced the frequency of micronuclei and aberrations of the chromosome in Chinese hamster’s ovary (CHO-K1) compared to control cell culture. Surprisingly, lower doses and shorter exposures were more effective in reducing the levels of these genotoxicity markers [37].

Yaday et al. cultured CHO-K1 cells with different types of CNPs, including MWCNTs, which induced alteration of the cell cytoskeleton. Elongation, an increase in the number of cytoplasmic vacuoles, and the formation of lamellipodia via actin polymerization were observed. Cytoskeleton remodeling was associated with enhanced expression of the Dlc-1, cofilin, and Rac1 proteins that affect the cytoskeleton and cell motility [38]. CHO-K1 was also influenced by GOs. Batiuskaite et al. found that GO (alone or with bovine serum albumin) significantly reduced cell viability in a dose-dependent manner. Importantly, GO-BSA induced lower changes in viability than GOs [39].

## 3. Reproductive Toxicity In Vivo Studies

In vivo studies determining the toxicity of CNPs used different types of animal models, both non-mammal and mammal species. Nematodes, insects, mice, and rats are the most commonly used species in nanotoxicity research. The results of most studies suggest that nanoparticles can damage the reproductive system and interfere with reproduction Table 1.

### 3.1. Experiments with Nonmammal Species

Nematodes are represented by *Caenorhabditis elegans* (roundworms, *C. elegans*) whose reproduction might be altered by various types of CNPs. Kim et al. exposed *C. elegans* to GOs at a dose of 10 mg/L. Two hours after exposure, GOs were detected throughout the body, including the reproductive system; however, at 48 h the GOs accumulated especially in the area around the germline and embryos. Furthermore, exposure to GOs altered spermatogenesis and decreased the number of sperm, thus inducing severe reproductive toxicity. It also altered fat metabolism and increased oxidative stress [40].

The reproductive toxicity of GOs was also described by Chatterjee et al. *C. elegans* was exposed to either GOs, reduced GOs, or both. GOs were uptaken by cells and accumulated in reproductive organs. A significant reduction in reproductive function was observed even after exposure to the low dose of 5 mg/L and the dose of 50 mg/L completely stopped reproduction. Exposure to GOs deregulated the MAPK (mitogen-activated protein kinase) and Wnt pathways. Interestingly, reduced GOs were more biocompatible and did not induce tissue to the damage of reproductive system [41].

Zhao et al. found that GO not only reduced reproductive capacity and altered gonad development in *C. elegans* but also induced germ cells. The signaling pathway involved in germ cell apoptosis was HUS-1/CLK-2-CEP-1-EGL-1-CED-4-CED-3, which reflects DNA damage and induction of apoptosis. GOs also activated miRNA 360 which interacts with the gene encoding CEP-1. In this way, GOs can alter the epigenetic signaling involved in the self-protection mechanism against GOs toxicity [42].

Kong et al. determined the toxicity of graphene and polylactic acid-functionalized graphene (PLA-G) on *C. elegans*. They exposed *C. elegans* to concentrations of 50–1000 µg/mL. In contrast to the results with GOs, graphene and PLA-graphene did not affect the reproductive capacity of *C. elegans*, indicating that the functionalization of CNPs may be beneficial and improve biocompatibility [43].

Other nonmammalian animal species for which the toxicity of CNPs was evaluated included insects (*Acheta domesticus*, *Spodoptera frugiperda, Drosophila melanogaster*, *Bombyx mori)* and aquatic animals (*Paracentrotus lividus*, *Anabas testudineus*). *Acheta domesticus*, cricket, was used in the study of Kapeta-Kaczmarek et al. who chronically exposed animals to different doses of nanodiamonds (NDs) in the diet of animals. Exposure to NDs decreased cricket survival (21 days of NDs vs. 28 days of control) and negatively influenced egg production and hatching success. The females in the higher NDs group laid an average of 15 eggs, the females in the lower dose 25 eggs, while the females in the control group laid 35 eggs in 48 h. The results indicate that exposure to NDs reduced fecundity [44].

*Spodoptera frugiperda*, fall armyworm, was exposed to different doses of oxidized MWCNTs and GOs in the diet. Martins et al. confirmed that, in a dose-dependent manner, both CNPs reduced fertility and fecundity [45]. Philbrook et al. exposed *Drosophila melanogaster* (*D. melanogaster*) fly, to hydroxylated single-walled carbon nanotubes that affected neither fertility nor fecundity [46]. However, a more recent study with *D. melanogaster* by Priyadarsiny et al. used GO nanosheets and revealed the toxic effect of CNPs. Especially teratotoxicity. In the hatching test, a significantly lower number of adult flies hatched from every vial. The decrease depended on the dose [47].

Fang et al. exposed *Bombyx mori* (*B. mori*) to GOs to evaluate its effect on reproduction. The dose of 25 mg/L of GOs induced oxidative stress and DNA damage in ovary cells and reduced the gonadosomatic index in *B. mori* larvae by 41%. GOs similarly increased the level of oxidative stress in silkworm ovary tissue, which was associated with a decrease in the number of both oogonia and oocytes in the ovary and increased vacuole formation in follicle cells. Transcription of genes related to ovarian development was also reduced [48].

Studies also evaluated the effect of CNPs on animals living near or in aquatic environments. Carbon black (CB) and GOs were tested for reproductive toxicity to *Paracentrotus lividus*, sea urchin. CB at doses of 0.0001–1.0 mg/L/h reduced egg fertilization by approximately 50%. On the other hand, GOs did not affect fertilization [49].

Sumi et al. focused on the effect of two sublethal doses of Buckminsterfullerene (BCF; 5 mg/L and 10 mg/L) for short- and long-term duration on *Anabas testudineus*, a freshwater fish. BCF reduced the weight of both the ovary and testes, the activity of antioxidant enzymes (SOD, GTP), and increased the production of ROS. Prolonged exposure was associated with histological alterations in the ovary and testes. In the ovary, atresia, vacuole formation, thickening of the vitellogenic oocyte membrane, or completely degenerated oocytes were detected. In the testes, the formation of vacuoles, the decrease in the number of sperm and spermatocytes, the distortion of the seminiferous epithelium, and atresia were found. The results indicate that BCF leads to reproductive toxicity [50].

The toxic effect of MWCNT–COOH was determined in adult *Danio rerio* (zebrafish). Arrillo et al. showed that doses of 0.5 and 1.0 ppm of MWCNT–COOH significantly increased oxidative stress and lipid peroxidation in their ovary and testicular tissue [51]. Zhao et al. used *Xenopus tropicalis* that was exposed to either 0.5 or 2.5 mg/L MWCNTs for 56 days. The presence of MWCNTs inhibited body growth, including gonads (testes and ovaries), and histopathological sample analysis revealed that spermatogonia and oocyte formation was negatively affected [52]. A pair of *Oryzias latipes* was injected once intraperitoneally with GO (25–200 μg/g) and the pair continued to breed for another 21 days (Dasmahapatra et al.). The dose-dependent reduction in fecundity was documented during the early days after injection. Furthermore, embryo hatchability was significantly reduced in the 200 µg/g group; but embryo mortality was not altered. Interestingly, folliculogenesis in the ovary and the morphology of granulosa and Leydig cells did not change significantly, although the authors identified GO agglomerates in the gonads [53].

### 3.2. Experiments with Mammals

Studies on mammals were conducted mainly in mice and rats.

Zhang et al. showed that graphene QD (GQD) administered by oral gavage or intravenously injected did not change sexual behavior, sperm quality, or testosterone levels in male mice. Even high doses are rapidly excreted from the body and did not accumulate in tissues. Female mice housed with males had first, second, and subsequent litters of healthy pups with no apparent differences from females housed with buffer-treated males [54]. Similar results were described by Skovmand et al. who intratracheally instilled GOs, amorphous CB (Flammruss 101), CB (Printex 90), and diesel particle matter (SRM1650b) in mice. Any changes in sperm parameters, sperm production, and testosterone levels were detected [55]. Liang et al., who administered GOs intravenously (25 mg/kg/d) to male mice, described that GO-treated mice did not show abnormalities in reproductive activity, hormonal levels, and sperm quality. Their offspring were also healthy and their survival rate and growth were similar to those of the control group. Furthermore, even a high intra-abdominal injection dose of 300 mg/kg in male mice (60 mg/kg/d for 5 days) did not cause damage to reproductive organs [56].

However, in contrast to the findings of the above studies, some studies show the opposite and describe a negative effect of CNPs on the reproduction of mice. For example, the study by Farshad et al. showed the toxic effect of SWCNTs and MWCNTs. BALB/c mice were orally administered 10 and 50 mg/kg/d for 5 weeks. Higher doses of SWCNTs significantly reduce body and testis, epididymis, and vas deferens weight, whereas MWCNT reduced only body weight. Both CNTs dose-dependently decreased sperm count, viability, and motility, and increased oxidative stress. Importantly, exposure to CNTs disrupted the mitochondrial functions of the sperm (elevated mitochondrial membrane depolarization and decreased dehydrogenase activity and ATP production. Histological analysis revealed testicular tissue injuries (tubular injury, tubular desquamation) and lowered spermatogenic index [57].

The toxic effect of nanoscale GO (NGOs) was evaluated by Akhavan et al. BALB/c mice were injected intravenously with different doses of NGOs (2, 20, 200, or 2000 µg/mL) and after 8 weeks were sacrificed. The NGOs were taken up by various types of tissue, including the thyroid and testes. Importantly, sperm viability and motility decreased dramatically in a dose-dependent manner from 75 to 40% and morphological abnormalities of the sperm tail and head were observed, especially at the highest dose. At doses of 200 µg/mL and 2000 µg/mL, ROS production in semen also increased and DNA fragmentation and aberrations occurred. Female mice inseminated by males treated with NGOs showed lower levels of follicle-stimulating hormone (FSH), luteinizing hormone (LH), progesterone, and prolactin during pregnancy. NGO concentration of 2000 µg/mL reduced the secretion of FSH, LH, progesterone, and prolactin by 38, 57, 31, and 37%. It was associated with altered fetus growth and a 15% reduction in postnatal viability of delivered pups. The results indicate that the NGOs altered reproductive health and even the next generations [58].

It seems that CNPs can negatively affect not only male mice, but also female mice. Hougaard et al. pre-conceptually administered MWCNTs (intratracheal instillation of 67 μg NM-400 MWCNT) to adult female mice. Subsequently, mice were bred together with adult males. The time to birth of the first litter was delayed by an average of 5 days. Interestingly, exposure to MWCNT caused lung and liver damage that lasted almost 4 months [59].

Johansson et al. evaluated the effect of intratracheally instilled MWCNTs on estrous cycle regularity and reproductive function. The administration of MWCNTs prolonged the estrous cycle during which exposure occurred. Before exposure, the estrous cycle lasted 3–5 days, after exposure, the estrous cycle increased by two days. In contrast, the cycle beginning after administration was shorter and lasted 4.3 days. Exposure to MWCNTs also affected delivery time. Mice exposed to the dose of 2 μg gave birth earlier compared to the control group, while the doses of 18 μg and 67 μg delayed delivery [60].

A similar effect was also documented for C_60_ (Buckminsterfullerene). The toxicity of C_60_ 1 µm diameter (micro-C_60_) and 50 nm diameter (nano-C_60_) in mice and rats was described in NTP Technical Report. Animals inhaled (nose only) C_60_ for 3 months. Micro-C_60_ at doses of 2, 15, or 30 mg/m^3^ decreased sperm motility in male mice and rats and increased the likelihood of a prolonged estrous cycle in female mice. Nano-C_60_ at concentrations of 0.5 or 2 mg/m^3^ lowered sperm motility in male rats and in female mice elevated the probability of an extended estrous cycle [64].

Nirmal et al. conducted two studies in which they intraperitoneally exposed rats to either three increasing doses of nanoscale GOs (NGOs; 0.4, 2.0, or 10.0 mg/kg) for 7, 15, or 30 days or hydroxylated MWCNTs at doses of 0.4, 2.0, and 10.0 mg/kg (15 doses). NGOs caused a dose-dependent reduction in sperm, spermatogonia, and spermatids. Furthermore, a decrease in sperm motility and morphological abnormalities (atrophy of seminiferous tubules with reduction in germinal epithelium, germ cells, and vacuolization) was detected in animals treated with the highest doses of NGOs [61]. OH–MWCNTs decreased sperm count and motility in a dose-dependent manner, viability was not affected; however, a significant increase in sperm abnormalities (headless sperms, absence of normal hook, amorphous head, bent tail, folded tails) was documented. Histological analysis revealed severe damage to testicular tissue. A dose of 2.0 mg/kg damaged the seminiferous tubules and induced vacuolization, caused interstitial engorgement and edema, and reduced the thickness of the germinal epithelium. More severe damage occurred after administration of the highest dose [62]. In summary, NGOs and MWCNTs had a similar destructive effect on the male reproductive system of rats.

Farombi et al. tested the response of pubertal rat organs to exposure to MWCNT–COOH. Rats were administered different doses of MWCNT–COOH suspension intraperitoneally (0.25, 0.5, 0.75, and 1.0 mg/kg/d) for 5 days. After treatment, the activity of antioxidant enzymes (superoxide dismutase and glutamate pyruvate transaminase) increased in the testes, epididymis, and sperm, as well as peroxide and malondialdehyde levels, while glutathione-S-transferase and glutathione levels decreased. Thus, oxidative stress increased significantly. Furthermore, MWCNT–COOH caused a decrease in the number and motility of sperm in the epididymis and the level of testosterone and an increase in sperm abnormalities and morphological changes in the testes and epididymis. The results indicate that MWCNT–COOH is toxic to the reproductive system of rats [63].

Both in vitro and in vivo studies show the toxic potential of CNPs, including grapheme, GO, MWCNTs, SWCNTs, QD, nanodiamonds, and fullerenes. They can alter spermatogenesis, sperm morphology and functions, hormonal balance, damaged ovary, and testicular tissue by inducing the production of reactive oxygen species, and DNA damage. However, in vivo studies also provided results that do not support reproductive toxicity of CNPs. The toxic effect depends on the type of CNPs, dose, and time of exposure.

## 4. Developmental Carbon Nanotoxicity

Developmental disorders and defects caused by xenobiotics depend on the toxicity to the parental organism, particularly the reproductive system, and on the transfer of xenobiotics through the placenta to the embryo or fetus. Developmental toxicity was tested mainly on aquatic animals (Table 2).

D’Amora et al. evaluated the toxicity of oxi-nano-onions (Oxi-CNOs), Oxi-nanohorns (Oxi-CNHs), and GOs to five-day *Danio rerio* embryos (zebrafish). Although Oxi-CNOs and Oxi-CNHs showed a fairly high level of biocompatibility, GOs in a dose-dependent manner altered hatching and development. Doses of 50 μg/mL and higher reduced the survival rate by 25%, delayed development, reduced heart rate, frequency of movement, and increased the rate of malformations including tail flexure, yolk sac, pericardial edema, etc. [65].

The toxicity of GOs to zebrafish embryos was also demonstrated by Yang et al. The dose of 10 μg/mL decreased the hatching rate, altered locomotor activity, increased malformation rate, and oxidative stress. Furthermore, GOs were responsible for the elevation of the expression of synapsin, neurogein1, α1-tubulin, sonic hedgehog protein (SHH), and rbl13 protein that are associated with neural development. Importantly, the mRNA levels of genes associated with immune response, such as interleukin-6 (IL-6), interleukin-8 (IL-8), tumor necrosis factor-α (TNF-α), and interferon-γ (IFN-γ), were significantly even under low environmental concentration exposure of GOs (0.01 μg/mL) [66].

The cardiovascular toxicity of GO in zebrafish embryos was documented by Bangeppagari et al. who exposed embryos to different doses of GO. Lower doses of 0.1–0.3 mg/mL did not affect development, while higher doses of 0.4–1 mg/mL resulted in delayed hatching and increased mortality and heartbeat. Severe cardiovascular defects with retardation of cardiac looping and reduced hemoglobinization were also detected [67]. Jaworski et al. compared the toxicity of pristine graphene and GOs in various models, including *D. rerio*, *Lemna minor*, HS-5 cells, and *Staphylococcus aureus*. GOs showed greater dose-dependent toxicity toward all living models. In zebrafish, the number of unhatched embryos increased, as well as the rate of developmental abnormalities, tail malformations, or pericardial edema. The concentrations of 50 and 100 μg/mL of both CNPs led to the death and coagulation of the embryos; therefore, the survival rate decreased [68]. The results of Chen et al. support the conclusions of the mentioned studies on the embryotoxicity of GO. They found that GO spontaneously penetrated the chorion and passed through endocytosis into the embryo and accumulated primarily in the eyes, yolk sac, and heart regions, and caused developmental defects associated with increased mitochondrial damage and ROS production [69].

In general, it is believed that the functionalization of CNPs may reduce their toxicity. Cao et al. used functionalized GO (GO–COOH) at doses of 10, 50, and 100 mg/L. Surprisingly, they found an elevated level of oxidative stress and disrupted development of the nervous system of zebrafish larvae, manifested as abnormalities in locomotor activity. Furthermore, acetylcholine esterase and ATPase activity and expression of genes related to Parkinson’s disease were up-regulated [70].

Falinski et al. and Martinez et al. tested the embryo and larval (zebrafish) toxicity of MWCNTs. Oxygenized MWCNTs increased embryonic mortality that did not depend on ROS levels. This suggests that not only oxidative stress but also the physicochemical properties of MWCNT play a key role in toxicity [71]. These results are consistent with a second study in which the toxic effect of short and long MWCNTs differed. While short MWCNTs reduced larval locomotor activity, altered neurodevelopment, and decreased neutrophil migration, long MWCNTs caused malformations, alteration of heart rhythm, and decrease in neutrophil migration [72].

Zhu et al. exposed D. rerio embryos (zebrafish) to C_60_ and fullerols. Fullerols did not exert direct toxicity on embryos at a dose of 50 mg/L after 96 h of exposure; however, a dose of 1.5 mg/L altered embryonal and larval development, delayed development, decreased hatching rate, and increased incidence of pericardial edema [73].

Other models of aquatic organisms, such as zebrafish, reflect the risks of potential toxicity and accumulation of CNPs in the aquatic environment that directly and indirectly affect humans. Martínez-Paz et al. evaluated the effect of MWCNTs on aquatic larvae of *Chironomus riparius*. The presence of MWCNTs caused a decrease in the expression of the gene responsible for DNA reparation, indicating that MWCNTs can activate apoptosis. However, the highest dose of MWCNTs (1000 μg/L) reduced the expression of the Hsp70 a and Hsp27 genes. These HSP (heat shock proteins) interact with apoptotic pathways and inhibit apoptosis [74]. Oxidized SWCNTs were tested by Zhu et al. on *Artemia salina*. The larvae were exposed to oxidized SWCNTs and a significant increase in mortality rate was found. After 24 h of exposure, body length and swimming speed also decreased in a dose-dependent manner. The swimming impairment was due to malformation of the gills and the attachment of oxidized SWCNTs to the gills. We cannot omit that SWCNTs also increased ROS production [75].

Mesarič et al. studied whether the exposure of sperm of *Paracentrotus lividus* affects the offspring. The embryos of eggs fertilized with sperm exposed to CNPs (carbon black or GO) showed abnormal and arrested development (irregular embryo shape, abnormal migration of primary mesenchymal cells, developmental delay and impaired skeletogenesis) and reduced cholinesterase activity in a dose-dependent manner [49].

Except for aquatic organisms, insects are a suitable animal model for evaluating the toxicity of CNPs. Liu et al. conducted a study with MWCNTs and Drosophila embryos. Of the embryos injected, nanoparticles penetrated the cells but did not reach the nucleus and remained in the cytoplasm. The MWCNTs caused the death of ectodermal but not neural stem cells. The results indicated that some cells are more susceptible to MWCNTs toxicity [76]. Dziewięcka et al. exposed *Acheta domesticus* to different doses of GOs in food and evaluated the negative multigenerational (three subsequent generations) effects of GOs. Even in the third generation, the changes induced by GOs were documented. GOs decreased the lifespan, reduced the number of larvae per female, and the hatching time was shorter in the first and third generations. Interestingly, the second generation did not show the same pattern of changes as the first and third generations [77].

Reproductive toxicity testing also includes studies in birds and mammals. Sawosz et al. evaluated the developmental toxicity of pristine graphene on chicken embryos. They exposed chicken fertilized eggs to different doses of pristine graphene (50–10,000 μg/L). After 19 days of incubation, embryo survival was significantly reduced, but biochemical parameters and body weight were unchanged. Brain samples showed an atypical ultrastructure with an increase in the number of vacuoles and vessels with accumulated leukocytes and mitochondrial damage. The expression of PCNA mRNA (proliferating cell nuclear antigen) at concentrations of 1000–10,000 μg/L was significantly decreased. PCNA is involved in DNA synthesis during the replication and post-replication DNA repair pathways [78].

Szmidt et al. compared the effect of three types of CNPs (pristine graphene, GOs, and reduced GOs) at different concentrations (50, 500, and 5000 μg/mL) on the chicken embryo. The CNP solutions were injected into the eggs and the incubation lasted for 18 days. Dose-dependently, the survival rate decreased significantly in all exposed groups (the highest negative effect was documented in the GO group). Liver samples showed that graphene caused the cell membrane to be disrupted, increased the number of vesicles, interrupted both the inner and outer mitochondrial membrane, and led to the degeneration of the mitochondrial cristae. Treatment with GOs did not damage the cell membrane but increased the number of intracellular vacuoles and severe mitochondrial damage was found in some hepatocytes. In the presence of reduced GOs, cytoplasmic disintegration and signs of mitochondrial fragmentation were observed. Interestingly, the levels of a marker of DNA damage, 8-hydroxy-2′-deoxyguanosine (8-OHdG), in the liver decreased in the groups treated with graphene and reduced GOs at concentrations of 50 and 500 μg/mL which indicated the antioxidant effect of these CNPs [79].

A study with six types of CNPs was performed by Kurantowicz et al. They injected solutions of nanodiamonds, graphite, pristine graphene, small GOs, large GOs, and reduced GOs (500 μg/L) into the egg albumin (chicken-embryo model). The relative survival rate decreased in all CNPs groups, except for nanodiamonds. The most profound impact on survival rate had large GOs. The presence of CNPs did not affect development, weight, liver, spleen, heart, brain, and kidney. The morphology of the erythrocytes and the levels of biochemical markers and oxidative stress did not differ among groups [80]. Jaworski et al. determined the hemocompatibility of CNPs and exposed fertilized chicken eggs to pristine graphene, GOs, and reduced GOs at concentrations of 50, 500, and 5000 μg/mL, respectively. In contrast to the results of Kurantowict et al., morphological changes in red blood cells were described. The cell membrane disintegrated, the cells became deformed (echinocytes, knizocytes), lost their biconcavity, and hemolysis occurred. The highest percentage of hemolysis was observed in the pristine graphene group at a concentration of 5000 μg/mL (73%). The presence of CNPs also improved the production of ROS [81].

Studies in mice have also been carried out which have confirmed some degree of toxicity of CNP, similar to the results of the studies mentioned above. Liu et al. orally administered pregnant mice with GO that caused dose-dependent complications, such as decreased dam and the live fetus, fetal skeletal malformations, increased number of resorbed embryos, and dead fetuses. The authors demonstrated that this pathological effect was mediated by an alteration of the gut microbiome, which was associated with impaired placental barrier function [82]. Fu et al. orally exposed lactating mice to GOs (0.5 mg/mL) which was associated with retardation of the increase of body weight and length, and length of the tail of filial mice, thus development was delayed. Interestingly, the organs (heart, liver, lung, kidney, spleen) of filial mice exposed to GOs exhibited severe atrophy; moreover, in filial mice receiving a high concentration of GOs, a reduction in intestinal villi length was detected [84].

Fujitani et al. who exposed pregnant mice intraperitoneally or intratracheally on the ninth day of gestation to different doses of MWCNTs (2, 3, 4, and 5 mg/kg). The rate of early fetal resorption increased in all groups. Intraperitoneal administration of MWCNTs resulted in fetal skeletal malformations (limb reduction, short or missing tail, and fused vertebrae) at all doses. These malformations also occurred in the offspring of mothers after intratracheal administration of MWCNTs; however, only the 4 and 5 mg doses induced these malformations [83].

Studies provide data suggesting that CNPs have the ability to cause developmental damage to larvae or fetus. CNPs harm both invertebrate and vertebrate offspring, including fish and mammals. They can reduce survival rates, slow down development, cause various types of malformations, and damage tissues, such as nervous, cardiac, liver, etc. The results are concerning, and more research is needed on developmental toxicity.

## 5. Conclusions

Studies conducted in vitro in various cell lines and in vivo in various animal models show that CNPs have toxic potential for the reproductive system and therefore may reduce fertility and, moreover, may affect the development of pups intrauterine and in later days or years of life. However, there is a significant difference between the toxicity of different CNPs and their types. The final toxic effect depends on the physicochemical characteristics of CNPs, the route of their administration (exposure pathway), the form of cellular uptake, the mechanisms of cellular toxicity, absorption, distribution, accumulation, degradation of CNPs in organisms, and excretion of CNPs from organisms. The main factors modulating interaction with organisms are size and surface area; surface properties, such as charge, hydrophilicity, and hydrophobicity; and shape, functionalization, and density of functional groups [85,86,87]. Dose, the period of time the cells or organisms are exposed to CNPs, also plays an important role in the intensity of toxicity. Higher doses and longer exposure times increase the toxic effect and the extent of damage.

The toxicity to the reproductive tract and developing fetus is often dependent on increased oxidative stress and DNA damage by CNPs.

CNPs interfere with sperm production, negatively affect sperm function, alter oocyte maturation, damage ovarian and testicular tissue, and affect hormone production (FSH, LH, prolactin, testosterone). These changes induced by the effects of CNP can significantly reduce fertility. CNPs that interact with the gravid female or directly with the fetus or larvae of animals can penetrate the fetus/larvae and cause permanent, and often very serious, damage to developing tissues, especially the skeletal, nervous, or cardiovascular system, and developmental retardation. It should also be mentioned that CNPs can reduce the survival rate.

In light of these facts, although some studies have not confirmed reproductive and developmental toxicity, it is necessary to conduct further studies on the toxicity of CNPs, including those involving humans, focusing specifically on reproductive and developmental toxicity to know exactly how nanoparticles affect the reproductive system and developing offspring.

## Figures and Tables

**Table 1:** Summary: in vivo nanotoxicity.

	CNPS	Exposure	Findings	References
*Caenorhabditis elegans*	GO	10 mg/L; 30 h	Accumulation in reproductive organs ↓ spermatogenesis ↑ ROS	Kim et al. [40]
	GO (single and few layers), pristine GNP, GNP-COOH, GNP-NH_2_	5, 50 mg/L; 72 h	Accumulation in reproductive organs ↓ reproduction potentiality SLGO > FLGO > GNP-pristine > GNP–NH_2_ > GNP–COOH	Chatterjee et al. [41]
	GO	25 µg/mL; 60 min	↑ germ cell apoptosis alteration of gonad development	Zhao et al. [42]
	GO; GNP, polylactic acid-GNP	50, 200, 500, 1000 μg/mL	No reproductive toxicity	Kong et al. [43]
*Acheta domesticus*	Nanodiamonds	20, 200 µg/g daily with food—until the death of the last individual.	↓ survival ↓egg production	Kapeta-Kaczmarek et al. [44]
*Spodoptera frugiperda*	MWCNTs, GO	0, 10, 100, 1000 µg/g; in diet	GO ↓ fertility and fecundity	Martins et al. [45]
*Drosophila melanogaster*	SWCNT-OH hydroxylated single-walled carbon nanotubes	0.005%, 0.01%, 0.05%, 0.1%, 0.5%, w/v	No reproductive toxicity	Philbrook et al. [46]
	GO	50, 100, 150, 200, 250, 300, and 500 mg/L; in food	dose of 500 mg/L flies lay much smaller number of eggs and do not turn into larvae ↑ death of ectodermal stem cells	Priyadarsiny et al. [47]
*Bombyx mori*	GO	25 mg/L	↑ ROS ↑ DNA damage ↓ reproduction ↓ oogonia and oocytes	Fang et al. [48]
*Paracentrotus lividus*	CB, GO	0.0001, 0.001, 0.01, 0.1, 1.0 mg/L; 1 h	CB at all doses ↓ reduction of egg fertilization 50%. GOs did not affect fertilization	Mesarič et al. [49]
*Anabas testudineus*	Fullerene	5 mg/L and 10 mg/L; 96 h or 60 days	↓ weight of the ovary and testes ↓ activity of antioxidant enzymes ↑ ROS 60 days = alteration of the ovary and testes ↓ number of sperm and spermatocytes	Sumi et al. [50]
*Danio rerio*	MWCNT–COOH	0.5 and 1.0 ppm; 48 h	↑ ROS + lipid peroxidation in ovary and testis	Carrillo et al. [51]
*Xenopus tropicalis*	MWCNT	0.5, 2.5 mg/L; 56 days.	↓ body growth ↓ gonads (testis, ovary)	Zhao et al. [52]
*Oryzias latipes*	GO	25, 50, 100, and 200 μg/g; intraperitoneal injection	Dose dependent ↓ fecundity ↓ embryo hatchability agglomerates of GO in gonads without tissue damage	Dasmahapatra et al. [53]
Mice	GQD	150 mg/kg, 500 μL/75 mg/kg, 200 μL oral gavage/intravenous injection	No reproductive toxicity	Zhang et al. [54]
	GO, amorphous CB (Flammruss 101), CB (Printex 90), and diesel particle matter (SRM1650b)	50 μL suspension intratracheally instilled	No reproductive toxicity	Skovmand et al. [55]
	GO	6.25–300 mg/kg Intraabdominal or intravenous application single or repeated administration	No reproductive toxicity	Liang et al. [56]
	SWCNTs MWCNTs	10, 50 mg/kg/d; for 5 weeks, orally administered	SWCNTs 50 mg/kg ↓ testis, epididymis, vas deferens both CNPs ↓ sperm count ↓ sperm viability and motility ↑ROS testicular tissue damage	Farshad et al. [57]
	nanoscale GO	2, 20, 200, 2000 µg/mL; injected intravenously	↓ sperm viability and motility morphological abnormalities of the sperm ↑ ROS production in semen + DNA fragmentation (200 µg/mL and 2000 µg/mL) Female mice inseminated by male NGOs ↓ FSH, LH, prolactin, progesterone during pregnancy	Akhavan et al. [58]
	MWCNTs	Total dose 67 μg animal; intratracheal application female mice were instilled once with 50 μL	mice bred together delayed time to birth of first litter	Hougaard et al. [59]
	MWCNTs	67 μg estrous cycle 2 μg,18 μg, 67 μg delayed delivery intratracheally instilled	↑ estrous cycle (2 days) 2 μg ↓ time to delivery 18 μg and 67 μg delayed delivery	Johansson et al. [60]
Rats	nanoscale GOs	0.4, 2.0, or 10.0 mg/kg for 7, 15, or 30 days	Dose dependent ↓ sperm count ↓ sperm motility ↑ morphological abnormalities in testicular tissue	Nirmal et al. [61]
	OH–MWCNTs	0.4, 2.0, and 10.0 mg/kg (15 doses)	Dose dependent ↓ sperm count ↓sperm motility ↑ sperm abnormities Severe testicular damage	Nirmal et al. [62]
	MWCNT–COOH	0.25, 0.5, 0.75, and 1.0 mg/kg/d for 5 days; intraperitoneal application	↑ activity of antioxidant enzymes and malondialdehyde level in the testes, epididymis and sperm ↓ sperm count ↓ sperm motility ↓ testosterone COOH caused ↑ sperm abnormities increase in sperm abnormalities morphological changes in the testes and epididymis.	Farombi et al. [63]

CB: carbon black; GNP: graphene; GNP–COOH: carboxylic acid graphene; GNP–NH_2_: graphene amid; GO: graphene oxide; rGO: reduced graphene oxide; GO–COOH: carboxylic acid functionalized GO; GQD: graphene quantum dots; MWCNTs: oxygenized multi-walled nanotubes; O–MWCNTs: oxygenized MWCNTs; OH–MWCNTs: hydroxylated MWCNTs; MWCNT–COOH: carboxylic acid MWCNT; ROS: reactive oxygen species; SWCNT–OH: hydroxylated single-walled carbon nanotubes; ↑↓ increase/decrease of the values, intensity or activity.

**Table 2:** Summary: developmental nanotoxicity.

	CNPS	Exposure Dose; Time	Findings	References
*Danio rerio*	Oxi-CNOs, Oxi-CNHs, GOs	5, 10, 50, 100 μg/mL/120 h after fertilization	↓ survival rate delayed development cardiotoxicity malformations	D’Amora et al. [65]
	GO	0, 0.01, 0.1, 1, 10 μg/mL; 2 hpf—5 days	↓ locomotor activity Malformation ↑ Oxidative stress	Yang et al. [66]
	GO	0.1–0.3, 0.4–1 mg/mL /14 h	cardiotoxicity ↑ mortality	Bangeppagari et al. [67]
	Pristine graphene, GOs	50 and 100 μg/mL /96 h	↑ mortality ↑ coagulation	Jaworski et al. [68]
	GOs	0.01, 1.0, 10, 100 mg/L/96 h	↓ hatching rate ↓ movement cardiotoxicity yolk sack edema eye damage	Chen et al. [69]
	GO–COOH	10, 50, 100 mg/L; 6–144 hpf	↓ locomotor activity ↓ tail coiling ↑ oxidative stress ↑ genes for acetylcholine esterase and ATPase neurotoxicity	Cao et al. [70]
	O–MWCNTs	50 μg/mL + 12.5, 25, 50 mg/L; 9 and 10.5 hpf—120 h	↑ ROS production ↑ mortality	Falinski et al. [71]
	Short + Long MWCNTs	0.005, 0.05, 0.5, 5, 50 ppm; 4 h post fertilization—2 days	↓ locomotor activity ↓ neutrophil migration + malformation cardiotoxicity ↓ neutrophil migration	Martines et al. [72]
	Fullerene and fullerenol	1.5 mg, 50 mg/L/1.5 hpf + 24–96 h	Fullerene ↓ development ↓ survival rate and hatching cardiotoxicity fullerenol—no toxic effect	Zhu et al. [73]
*Chironomus riparius*	MWCNT–COOH	10, 100, 1000 μg/L/24 h	↓ expression of Hsp27, Hsp70 ↑ relative RNA expression	Martínez-Paz et al. [74]
*Artemia salina*	O–SWCNTs	0–600 mg/L; 24 h	↑ mortality ↑ oxidative stress ↓ locomotor activity ↓ body length	Zhu et al. [75]
*Paracentrotus lividus*	Carbon black or GO	0.0001–1.0 mg/L; sperm exposed to GO	↓ cholinesterase activity morphological abnormalities	Mesarič et al. [49]
*Drosophila* embryos	MWCNTs	5 pg /embryo/1 injection	no toxicity, expect ↑ death of ectodermal stem cells	Liu et al. [76]
*Acheta domesticus*	GO	0.2, 2, 20 μg/g/1 injection	↓ lifespan ↓ number of larvae ↓ hatching time Changes to 3rd generation	Dziewięcka et al. [77]
*Chicken embryos*	pristine GPN	50–10,000 μg/L (1000–10,000); injection in ovo	↓ survival rate ↓ PCNA expression neurotoxicity vascular toxicity	Sawosz et al. [78]
	pristine GPN, GO, rGO	50, 500, 5000 μg/mL; injection in ovo	↓ survival rate liver toxicity DNA damage	Szmidt et al. [79]
	nanodiamonds, graphite, pristine GPN, small GO, large GO, rGO	500 μg/l; injection in ovo	No reproductive toxicity	Kurantowicz et al. [80]
	GO	50, 500, 5000 μg/mL; injection in ovo	↑ ROS hematotoxicity	Jaworski et al. [81]
Pregnant mice	GO	0.2 mL/10 g body weight daily during organogenesis perion	↑dead fetus ↑resorb embryos skeletal malformations microbiome disruption	Liu et al. [82]
	MWCNTs	2, 3, 4, 5 mg/kg body weight; intraperitoneal injection, 9 gestational days vs. 3, 4, 5 mg/kg body weight; intratracheal application	↑ fetal resorption skeletal malformation	Fujitani et al. [83]
Lactating mice	GO	0.05, 0.5 mg/mL; drinking water/21 days	↓ development (weight, length) organ toxicity	Fu et al. [84]

hpf: hours after fertilization; Oxi-CNOs: oxi-nano-onions; Oxi-CNHs: oxi-nanohorns; GO: graphene oxide; rGO: reduced graphene oxide; GO–COOH: carboxylic acid functionalized GO; GPN: graphene; MWCNTs: oxygenized multi-walled nanotubes; O–MWCNTs: oxygenized MWCNTs; MWCNT–COOH: carboxylic acid MWCNT; O–SWCNTs: oxygenized single-walled nanotubes; ↑↓ increase/decrease of the values, intensity or activity.

## Data Availability

Not applicable.
